# Multimodal AI Markers for aMCI Detection: Integrating Language, Speech, and Facial Analysis in an Argentine Cohort

**DOI:** 10.1002/alz70857_100061

**Published:** 2025-12-24

**Authors:** Greta Keller, Laouen Belloli, Lara Gauder, Agostina Carello, Ricardo Allegri, Luciana Ferrer, Diego Fernandez Slezak, Luc¡a Crivelli

**Affiliations:** ^1^ Fleni, Buenos Aires, Argentina; ^2^ University of Buenos Aires, Ciudad Autonoma de Buenos Aires, CABA, Argentina; ^3^ Institute of Neurosciences (INEU), Fleni‐CONICET, Buenos Aires, Buenos Aires, Argentina; ^4^ Institute of Computer Science, University of Buenos Aires, Buenos Aires, Argentina; ^5^ National Council for Scientific and Technical Research (CONICET), Buenos Aires, Argentina

## Abstract

**Background:**

The early detection of amnestic mild cognitive impairment (aMCI) is essential for effective preventive interventions. Artificial Intelligence (AI) offers innovative methods to identify markers of aMCI, complementing traditional approaches. However, research in this field remains in its early stages. In this context, this study focuses on designing AI multimodal neuropsychological instruments to differentiate healthy individuals from aMCI subjects, emphasizing local data calibration and validation.

**Method:**

We recruited 59 participants from Fleni, Argentina, including 30 healthy controls and 29 individuals diagnosed with aMCI based on Petersen's criteria (2020). All participants underwent comprehensive assessments, including neuropsychological testing (Uniform Data Set 3) and magnetic resonance imaging (MRI) following the ADNI 3 protocol. Participants were video‐ and audio‐recorded while performing language tasks on a web platform, which involved describing two target images (“Cookie Theft” and “Firefighter‐Oasis”) and completing two additional tasks without images (describing their favorite sandwich and reading a story). Multimodal markers were extracted from five modalities: language processing (automated speech transcription), speech acoustics (audio), face mesh analysis (video), blend shapes (video), and emotion recognition (video). Each modality provided a variety of features, including expert‐derived metrics and embedding representations. These features were used to train machine learning classifiers to differentiate individuals with aMCI from healthy controls.

**Result:**

Participants ranged in age from 60 to 89 years (mean ± SD: 70.95 ± 6.8). Unimodal analysis was performed to study shared information between proposed AI‐markers and traditional neurocognitive tests. We obtained 204 significantly correlated AI‐markers to traditional tests of a total of 432 (47%). Univariate AUC for aMCI diagnosis was measured for all markers, yielding an average above chance performance (0.57 ± 0.062). However, combining all modalities using a multivariate random forest classifier achieved an outstanding AUC of 0.91, highlighting its excellent diagnostic performance.

**Conclusion:**

This study demonstrates that AI‐based multimodal markers, including language, speech acoustics, facial analysis, and emotion recognition, can effectively differentiate aMCI from healthy controls in an Argentine population. Validating these tools using Spanish‐language data and cost‐effective, non‐invasive methods is crucial for their broader applicability.

